# Causes of mortality and severe morbidity in young children in Sierra Leone: a prospective cohort study

**DOI:** 10.1016/j.langlo.2026.104025

**Published:** 2026-08-10

**Authors:** Kwabena Owusu-Kyei, Maria Cussó, Augustin Eric Fombah, Llorenç Quintó, Andreu Bofill, Alba Morató, Victoria Fumadó, Mohamed Samai, Clara Menéndez, ICARIA Safety Team, Gerard Martinez, Celia Martí, María Cussó, Emma Wilheim, María Iriondo, Laia Nou, Noelia Arguedas, María Suay, Paula Sevilla, Laura Pellicer, Ariadna Rubio, Clara Izquierdo, Maria Goretti López, Victoria Fumadó

**Affiliations:** a Barcelona Institute for Global Health, Hospital Clinic-University of Barcelona, Barcelona, Spain; b Facultat de Medicina i Ciències de la Salut, Universitat de Barcelona, Barcelona, Spain; c College of Medicine and Allied Health Sciences, University of Sierra Leone, Freetown, Sierra Leone; d Hospital Sant Joan de Déu, Barcelona, Spain; e Centro de Investigação em Saúde de Manhiça, Maputo, Mozambique; f Investigación Biomédica en Red de Epidemiología y Salud Pública, Madrid, Spain

## Abstract

**Background:**

Accurate data on morbidity and mortality in children younger than 2 years remain scarce in many low-income and middle-income countries. We aimed to examine the causes and risk factors for severe morbidity and mortality in young children in Sierra Leone.

**Methods:**

We conducted a prospective cohort study nested within a randomised controlled trial (NCT04235816). The study was done in 14 primary health facilities in three districts in Sierra Leone. We enrolled infants aged 6–10 weeks, eligible for pentavalent vaccine dose 1 through the EPI programme, weighing at least 2·5 kg, resident in the study area, without known contraindications to macrolides or sulfadoxine–pyrimethamine, between March 17, 2021, and April 25, 2024. The objective was to assess causes of hospital admission and death through to Dec 31, 2024, when approximately 80% of children had completed 18-month follow-up. Hospital admissions, deaths, and immunisation status were captured through facility surveillance, household visits, telephone follow-ups, and community informants. Causes of death were classified using ICD-10 and the WHO 2016 verbal autopsy tool (InSilicoVA). Incidence rates were expressed per 1000 child-months at risk. Morbidity was analysed using negative binomial regression; in-hospital mortality was analysed using penalised logistic regression, and post-discharge mortality was analysed using Cox proportional hazards regression.

**Findings:**

Data on severe morbidity and mortality were collected from 20 560 children, of whom 10 644 (52%) were male and 9916 (48%) were female; the most common ethnicity was Temne (14 048 [68%] of 20 551 children with available data). Median follow-up was 16·6 months (IQR 16·0–16·7). There were 2688 hospital admissions (excluding readmissions and in-hospital deaths), yielding an incidence of 8·67 per 1000 child-months at risk (95% CI 8·35–9·00). Malaria (1459 [54·3%] of 2688 admissions), lower respiratory tract infections including pneumonia (449 [16·7%]), diarrhoea (259 [9·6%]), undernutrition (198 [7·4%]), and sepsis (128 [4·8%]) were the leading causes of admission. The leading causes of in-hospital deaths were malaria (122 [50·6%] of 241), lower respiratory tract infections (41 [17·0%]), sepsis (33 [13·7%]), undernutrition (17 [7·1%]), and diarrhoea (nine [3·7%]). The leading causes of community deaths for which a verbal autopsy was performed were malaria (103 [24·2%] of 425), lower respiratory tract infections (76 [17·9%]), other infectious diseases (73 [17·2%]), and diarrhoea (54 [12·7%]). Post-discharge mortality was 33·8 per 1000 child-months at risk (95% CI 27·08–42·20). Being underweight was associated with higher in-hospital mortality compared with not being underweight (adjusted odds ratio [OR] 1·60 [95% CI 1·21–2·12]). Full immunisation was associated with lower overall mortality (adjusted hazard ratio [HR] 0·65 [95% CI 0·55–0·76]), lower in-hospital mortality (adjusted OR 0·71 [95% CI 0·54–0·95]), and lower post-discharge mortality (adjusted HR 0·56 [95% CI 0·33–0·92]).

**Interpretation:**

This study shows that despite major investments and implementation efforts, mortality in infants and young children in Sierra Leone remains high and predominantly caused by preventable infections. These findings highlight the substantial unfinished agenda in global health and the urgent need for more effective tools and strategies.

**Funding:**

Gates Foundation and La Caixa Foundation.

## Introduction

The progress in global child mortality reduction achieved by 2015^1^ has stalled and an estimated 4·9 million deaths in children younger than 5 years occurred in 2024, with infectious diseases being the leading causes of mortality after the neonatal period.^1^ Of these deaths, 2·6 million were in children aged 1–59 months.^1^

**Research in context**

**Evidence before this study**

We searched PubMed, Embase, and the WHO Global Health Library for articles published from database inception to Dec 31, 2024, using combinations of the terms “low- and middle-income countries”, “under-two mortality”, “under-five mortality”, “post-discharge”, “sub-Saharan Africa”, and “Sierra Leone”, with no language restrictions. Previous studies from sub-Saharan Africa show that the majority of deaths in children younger than 5 years are attributable to preventable infectious diseases—particularly malaria, pneumonia, and diarrhoea—and that the post-discharge period is a time of high mortality risk. No previous prospective cohort study has combined health facility surveillance, household visits, telephone follow-ups, and community informant reports to provide a comprehensive description of early childhood survival patterns in this setting.

**Added value of this study**

Despite global progress in reducing child mortality, Sierra Leone continues to have some of the highest mortality rates for children younger than 5 years in the world. This persists despite substantial investments in child health, highlighting the need for more targeted and effective interventions. Evidence to guide such interventions remains limited, particularly regarding the specific causes of death and patterns of hospital admissions in children younger than 2 years. National data are often incomplete, hospital records sparse, and community-level mortality surveillance minimal. To our knowledge, this study provides the first comprehensive, systematic assessment of the main causes of death and hospital admissions in children younger than 2 years in Sierra Leone. By analysing hospital records and community-level data, we quantified the relative contributions of infectious diseases and other crucial health challenges in this population. Our findings offer actionable insights to inform health policy, guide resource allocation, and tailor interventions to reduce early childhood mortality. Importantly, this study addresses an essential knowledge gap, highlighting the persistent challenges in child survival and underscoring the unfinished global health agenda for young children in high-burden settings.

**Implications of all the available evidence**

Together with previous regional evidence, our findings show that infectious diseases, particularly malaria, respiratory infections, diarrhoeal illnesses, and sepsis, remain the leading causes of hospital admissions and deaths for young children, most of which were potentially preventable. Complete immunisation showed a strong protective effect, reinforcing the value of universal vaccination as a cost-effective child survival strategy. High-risk groups, including readmitted and undernourished children, require targeted interventions, such as strengthened discharge planning, community follow-up, and nutritional support. The notable post-discharge mortality further underscores the need for systematic post-discharge care, integrating community health worker engagement, follow-up protocols, and caregiver education on early recognition of danger signs.

WHO recommends perennial malaria chemoprevention with sulfadoxine–pyrimethamine for infants in areas of high–moderate malaria transmission.^2^ Sierra Leone adopted perennial malaria chemoprevention in 2018 and the malaria vaccine in 2024.^3^^,^^4^ Increasing the coverage of both strategies—which, in Sierra Leone, is currently 54% for the full six doses of sulfadoxine–pyrimethamine and 21% for the full malaria vaccine regimen—will require substantial efforts.^5^^,^^6^ Post-discharge malaria chemoprevention, a strategy recommended to reduce mortality in children admitted with severe anaemia in malaria-endemic settings, is not implemented in Sierra Leone.^7^

Despite the major setbacks of a 11 years of civil war and the Ebola virus disease outbreak, which severely affected the health system in Sierra Leone,^8^ the country has launched relevant health initiatives, such as the Free Health Care Initiative, which removes user fees for drugs and treatment at public health facilities for pregnant and breastfeeding women and children younger than 5 years.^9^ However, the implementation of this initiative is likely to fall short in adequately reaching the most vulnerable populations, which is reflected in the high mortality in infants (55 per 1000 livebirths) and children younger than 5 years (91 per 1000 livebirths).^10^

Child mortality surveillance systems reporting indicators using aggregated estimates for children younger than 5 years can obscure crucial, age-specific, epidemiological patterns. In Sierra Leone, extracting morbidity and mortality data for children younger than 2 years from the District Health Information Software 2 is difficult owing to the absence of age-disaggregated data within the broader category of children younger than 5 years.^11^^,^^12^

The primary aim of this study was to identify the leading causes and risk factors of severe morbidity and mortality in children younger than 2 years in Sierra Leone. We aimed to further assess the effect of routine immunisation and analyse the geospatial distribution of deaths in this age group to optimise the targeting and effect of health interventions.

## Methods

### Study design and participants

This prospective cohort study was conducted in Sierra Leone, where malaria transmission is perennial with seasonal peaks from June to August; in children younger than 5 years, the last average malaria prevalence in the 2021 Malaria Indicator Survey was 39% by microscopy.^13^ Malaria control strategies in the country are based on the use of long-lasting insecticidal nets, treatment with artemisinin-based combination therapy, perennial malaria chemoprevention, and the malaria vaccine.

Average national HIV prevalence in adults aged 15–49 years is 1·4%,^14^ and tuberculosis incidence is 283 per 100 000 population.^15^ Recurrent outbreaks of emerging infections strain the health system.

This study was conducted in three districts in the northern and northwestern provinces of Sierra Leone—Bombali, Tonkolili, and Port Loko.^16^ The study included 14 primary health facilities: four government-run hospitals, nine government-run peripheral health units, and one private clinic, covering both urban and rural areas. Although government health facilities provide free medical care for children younger than 5 years, frequent stockouts of essential medications, such as antimalarials, occur.

The study protocol and informed consent forms were approved by the Sierra Leone Ethics and Scientific Review Committee (dated Aug 9, 2020, no approval number) and the Hospital Clínic Research Ethics Committee (Barcelona, Spain; registration number HCB/2020/0173, dated Aug 28, 2020). The study adhered to the Declaration of Helsinki. The randomised controlled trial in which this study was nested was registered with ClinicalTrials.gov, NCT04235816, on Jan 17, 2020.

The study was done in children enrolled in a clinical trial (ICARIA), which evaluated the effect of oral azithromycin on child mortality in Sierra Leone. Children enrolled in the trial were individually randomly assigned to receive either oral azithromycin or placebo at scheduled Expanded Programme on Immunization (EPI) contacts; therefore, there were no separate control communities receiving a different community-level intervention. This analysis was conducted while the trial remained blinded. 20 560 infants aged 6–10 weeks, eligible for pentavalent vaccine dose 1 through the EPI programme, weighing at least 2·5 kg, resident in the study area, without known contraindications to macrolides or sulfadoxine–pyrimethamine, and whose parents or guardians provided written informed consent, were enrolled from March 17, 2021 to April 25, 2024; by Dec 31, 2024, approximately 80% of children had completed end of study follow-up at age 18 months. Infants were enrolled when presenting for the pentavalent vaccine dose 1 visit through the EPI programme at participating health facilities.

### Procedures

Information on participants' ethnicity, routine immunisation status, morbidity, and mortality was reported by participants' caregivers or collected from clinical records and child health cards. Participants' sex was recorded directly from the child health card by study staff. Each participant was issued a unique study card for identification. Information on children’s attendance at outpatient clinics and hospital admissions was recorded by study staff through daily visits to project health facilities plus telephone calls, household visits, and notifications from local key informants.

For hospital admissions and in-hospital deaths with clinical records, diagnoses were assigned through review of the available medical documentation, including the attending physician’s recorded diagnosis. Two study physicians independently reviewed the clinical records and assigned a primary diagnosis using the ICD-10. Cases with disagreement were discussed with a third clinician and final diagnosis was assigned by consensus. In case of deaths at home and in-hospital deaths with incomplete or without clinical records, data were gathered using the 2016 WHO Verbal Autopsy instrument,^17^ whereby trained study nurses conducted interviews with caregivers of deceased children using standardised questionnaires to obtain in-depth information on clinical history, symptoms, and circumstances leading to death. Training covered questionnaire administration, culturally appropriate interviewing, probing techniques, and data quality procedures. Data were collected electronically using structured forms with built-in checks and were reviewed for completeness and internal consistency before cause-of-death assignment using the InSilicoVA software.^18^ This tool was selected for cause-of-death assignment because it provides a standardised probabilistic approach and incorporates uncertainty in the attribution process (certainty was high in 94% of cases, moderate in 6%, and low in none).

Malaria diagnosis in hospital admissions and in-hospital deaths with clinical records was based on malaria rapid diagnostic tests and microscopy when available. In community deaths and in-hospital deaths without clinical records, malaria cause-of-death assignment was based on verbal autopsy.

### Statistical analysis

Baseline characteristics of study participants were summarised using descriptive statistics. A child was considered fully immunised for age if they had received all vaccines scheduled in the EPI programme up to their age at the relevant timepoint. A child was considered partially immunised if they had received at least one, but not all, vaccines due for their age, and non-immunised if they had received no vaccines. The EPI schedule in Sierra Leone included vaccines administered at birth (Bacillus Calmette-Guérin [BCG] and oral polio vaccine [OPV] dose 0), age 6 weeks (pentavalent vaccine dose 1, OPV dose 1, pneumococcal conjugate vaccine [PCV] dose 1, and rotavirus vaccine dose 1), age 10 weeks (pentavalent vaccine dose 2, OPV dose 2, PCV dose 2, and rotavirus vaccine dose 2), age 14 weeks (pentavalent vaccine dose 3, OPV dose 3, and PCV dose 3), age 9 months (measles–rubella and yellow fever), and age 15 months (measles booster). Nutritional status was established using the weight-for-age Z score, calculated in Stata.^19^^,^^20^

Sepsis was defined clinically as a life-threatening organ dysfunction of the respiratory, cardiovascular, coagulation, or neurological systems, in children with suspected or confirmed infection.^21^ Blood cultures were not performed in any of the children with suspected sepsis due to a lack of laboratory capacity. Lower respiratory tract infections were defined as acute infections of the lower respiratory tract, diagnosed on the basis of respiratory symptoms and signs, and, where available, chest x-ray.^22^

Person-time at risk was computed from the date of recruitment to the study until the earliest of: completion of the 18-month follow-up visit, death, study withdrawal, or the analysis date set at Dec 31, 2024. Incidence rate was expressed as episodes per 1000 child-months at risk (1 child-month at risk=30·4 days). The following exclusions were applied in the analysis: time spent during hospitalisation; a 7-day exclusion window after discharge (lag-period); and readmissions within the same ICD-10 chapter during this period, which were classified as non-incident. This window was selected as a threshold to capture continuation or early recurrence of the same clinical episode after discharge and was supported by the empirical distribution of event duration in the cohort, as 7 days approximated the 90th percentile of serious adverse event duration.

Follow-up time was split into non-overlapping intervals, during which key time-varying covariates remained constant. Immunisation status was defined as age-appropriate and modelled as a time-varying exposure. Follow-up time was split at age thresholds in the EPI schedule and at dates of vaccine receipt. Splits occurred at seasonal transitions (May 1 and November 1, reflecting the rainy and dry seasons, respectively), age categories aligned with the national EPI schedule, and dates of receipt of age-specific vaccines and sulfadoxine–pyrimethamine doses. Additional splits were generated for hospitalisations to account for lag and post-discharge risk periods.

Post-discharge mortality was defined as any death occurring within 28 days after hospital discharge. As a sensitivity analysis, post-discharge mortality was alternatively defined as death occurring within 14 days after hospital discharge. We assessed whether the cause of death recorded at the time of death matched the discharge diagnosis from the immediately preceding admission. Causes of death assigned by the InSilicoVA software were not included in the code-level comparison because this method might introduce errors in cause of death attribution.

All-cause mortality and post-discharge mortality were analysed using Cox proportional hazards regression. The proportional hazards assumption was assessed using Schoenfeld residuals,^23^ and child age in months was used as the underlying time scale; therefore, age was not included as a separate covariate. Follow-up began at enrolment and ended at death, withdrawal or loss to follow-up, or completion of follow-up. In-hospital mortality was modelled using penalised logistic regression^24^^,^^25^ with standard errors estimated by 1000 cluster bootstrap replications at the individual level. Negative binomial regression with clustering by child was used to analyse severe morbidity. Categorical predictors with more than two levels were coded according to the research question: dummy coding for comparisons against a reference and effect coding for deviations from the mean. Results were presented as hazard ratios (HRs), odds ratios (ORs), or incidence rate ratios (IRRs) with 95% CIs. Adjustment variables were selected a priori based on epidemiological relevance and literature on determinants of child mortality in sub-Saharan Africa.^16^^,^^26^, ^27^, ^28^, ^29^ For overall mortality, adjustment was based on cohort-level demographic and time-varying exposure variables, whereas for in-hospital and post-discharge mortality, additional admission-related variables (sex, district of residence, vaccination status, season of event, and nutritional status) were included where relevant. Season was defined according to the timing of the event or risk period during follow-up.

We did an analysis of malaria-related hospital admissions using negative binomial regression and a malaria-specific mortality analysis using Fine and Gray competing-risks regression. To assess the potential effect of deaths with unknown cause on malaria-specific mortality estimates, we conducted two sensitivity analyses: a plausible scenario in which 33% of deaths with unknown cause were randomly reclassified as malaria deaths, reflecting the proportion of malaria among deaths with known cause, and a worst-case scenario in which deaths with unknown cause were reclassified as malaria deaths.

For geospatial analyses, distance from each participant’s residence to the nearest health facility was estimated as road-network routing distance using the OpenRouteService application programming interface, and outputs were checked and mapped in QGIS.^30^^,^^31^ Mortality was mapped by 2 km grid cells as the proportion of deaths in enrolled children residing in each cell. All statistical analyses were performed using Stata SE version 19.

### Role of the funding source

The funders of the study had no role in study design, data collection, data analysis, data interpretation, or writing of the report.

## Results

Between March 17, 2021, and Dec 31, 2024, data on severe morbidity and mortality were collected from 20 560 children, of whom 10 644 (52%) were male and 9916 (48%) were female; the most common ethnicity was Temne (14 048 [68%] of 20 551 children with available data). There were 2936 hospital admissions in 2517 children and 859 deaths. Of these deaths, 345 (40%) occurred in hospital and 514 (60%) occurred at home (figure 1). The median age at recruitment was 6 weeks (IQR 6–7), and the median follow-up was 16·6 months (IQR 16·0–16·7). The estimated cumulative probability of death by age 12 months in enrolled children was 31·3 per 1000 (95% CI 29·0–33·8). Participant follow-up for this analysis is shown in figure 1.

**Figure 1:**
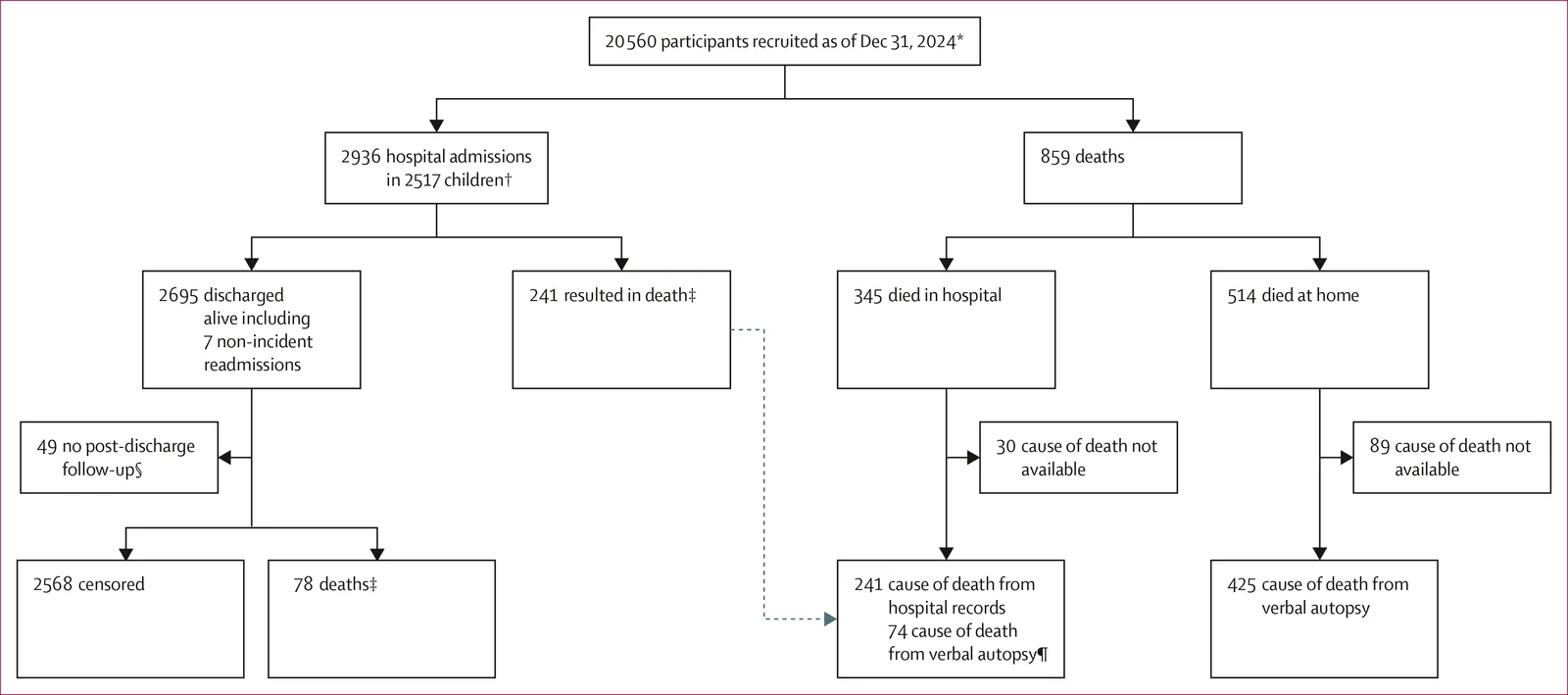
Study flowchart ∗16 436 completed follow-up. †Including hospital deaths. ‡Included in total deaths. §Participants' follow-up ended during or immediately after hospitalisation. ¶Deaths before hospital admission.

The median time from death to verbal autopsy interview was 59 days (IQR 29–111). The respondent was the parent or caregiver or another adult most knowledgeable about the child’s terminal illness and circumstances preceding death.

After excluding readmissions and in-hospital deaths, 2688 non-fatal hospital admission episodes were included in the analysis (figure 1), corresponding to an incidence of admission of 8·67 per 1000 child-months at risk (95% CI 8·35–9·00). The most common diagnoses in hospitalised children were malaria (1459 [54·3%] of 2688 admissions); lower respiratory tract infections, including pneumonia (449 [16·7%]); diarrhoea (259 [9·6%]); undernutrition (198 [7·4%]); and sepsis (128 [4·8%]; table 1). The incidence of admission was lower in female children than in male children (adjusted IRR 0·80 [95% CI 0·73–0·87]; p<0·0001), and in the rainy season compared with the dry season (0·89 [0·82–0·96]; p=0·0037; table 2). There was no difference in hospitalisation incidence rate in children who were fully immunised for age compared with those who were not (1·00 [0·90–1·12]; p=0·97; table 2). Regarding place of residence, the highest incidence of admission was in Tonkolili district (1·67 [1·56–1·78]) and the lowest in Port Loko (0·72 [0·67–0·77]) compared with the grand mean (p<0·0001). Age-disaggregated distributions of the leading causes of admission and death are shown in appendix 1 (p 1).

**Table 1:** Primary diagnoses of non-fatal hospital admission episodes

	Total non-fatal hospital admission episodes (n=2688)
Malaria	1459 (54·3%)
Lower respiratory tract infections (including pneumonia)	449 (16·7%)
Diarrhoea	259 (9·6%)
Undernutrition	198 (7·4%)
Sepsis	128 (4·8%)
Injuries	73 (2·7%)
Other infectious diseases	57 (2·1%)
Other non-infectious diseases	28 (1·0%)
Meningitis	11 (0·4%)
Measles	10 (0·4%)
Tuberculosis	3 (0·1%)
HIV/AIDS	2 (0·1%)
Unspecified	11 (0·4%)

Data are n (%).

**Table 2:** Factors associated with hospital admissions

	Rate estimates				Crude models∗			Adjusted model∗		
	Participants	Hospital admissions	Person-months	Incidence rate† (95% CI)	IRR (95% CI)	p value	Overall p value	IRR (95% CI)	p value	Overall p value
Age										
<6 months	20 559	730	90 920·87	8·03 (7·47–8·63)	1 (ref)	··	<0·0001	1 (ref)	··	<0·0001
6 months to <12 months	20 129	1175	118 086·05	9·95 (9·40–10·54)	1·27 (1·15–1·41)	<0·0001‡	··	1·28 (1·15–1·42)	<0·0001‡	··
≥12 months	18 481	783	101 143·52	7·74 (7·22–8·30)	0·98 (0·88–1·09)	0·70‡	··	1·00 (0·89–1·12)	0·94‡	··
Sex										
Male	10 644	1532	160 589·62	9·54 (9·07–10·03)	1 (ref)	··	··	1 (ref)	··	··
Female	9916	1156	149 560·82	7·73 (7·30–8·19)	0·81 (0·74–0·88)	<0·0001‡	··	0·80 (0·73–0·87)	<0·0001‡	··
District										
Port Loko	7433	727	110 808·80	6·56 (6·10–7·06)	0·72 (0·68–0·77)	<0·0001§	<0·0001	0·72 (0·67–0·77)	<0·0001§	<0·0001
Tonkolili	4259	938	63 999·29	14·66 (13·75–15·63)	1·66 (1·55–1·77)	<0·0001§	··	1·67 (1·56–1·78)	<0·0001§	··
Bombali	8868	1023	135 342·35	7·56 (7·11–8·04)	0·84 (0·79–0·89)	<0·0001§	··	0·83 (0·78–0·88)	<0·0001§	··
Season										
Dry	20 426	1383	150 087·55	9·21 (8·74–9·71)	1 (ref)	··	··	1 (ref)	··	··
Rainy	20 404	1305	160 062·89	8·15 (7·72–8·61)	0·88 (0·82–0·96)	0·0019‡	··	0·89 (0·82–0·96)	0·0037‡	··
Fully immunised¶										
No	20 414	677	81 807·29	8·28 (7·68–8·92)	1 (ref)	··	··	1 (ref)	··	··
Yes	20 048	2011	228 343·16	8·81 (8·43–9·20)	1·07 (0·97–1·17)	0·16‡	··	1·00 (0·90–1·12)	0·97‡	··
Sulfadoxine–pyrimethamine complete||										
No	20 446	1038	122 812·86	8·45 (7·95–8·98)	1 (ref)	··	··	1 (ref)	··	··
Yes	20 435	1650	187 337·58	8·81 (8·39–9·24)	1·04 (0·96–1·13)	0·32‡	··	1·04 (0·94–1·16)	0·41‡	··
Total	20 560	2688	310 150·44	8·67 (8·35–9·00)	··	··	··	··	··	··

Adjusted estimates are derived from a multivariable model including all covariates presented in the table. Season was defined according to month of hospital admission or death (rainy season: May–October; dry season: November–April). p values of 0·05 or lower are considered statistically significant. IRR=incidence rate ratio.

∗ Negative Binomial Regression.

† Per 1000 person-months at risk.

‡ Coefficients compared as difference from the reference (base) level.

§ Coefficients compared as difference from the grand mean.

¶ Fully immunised: all age-eligible Expanded Programme on Immunization vaccines received.

|| Sulfadoxine–pyrimethamine complete: all age-eligible sulfadoxine–pyrimethamine doses received.

In an analysis restricted to malaria-related admissions, older age was associated with a higher incidence of malaria-related hospitalisation, whereas female sex and the rainy season were associated with a lower incidence compared with male sex and the dry season. No association was observed with full immunisation per age, sulfadoxine–pyrimethamine uptake, or district of residence (appendix 1 p 4).

There were 859 deaths, with a mortality of 2·77 per 1000 child-months at risk (95% CI 2·59–2·96). Of the 241 in-hospital deaths with medical records, the most common causes of death were malaria (122 [50·6%]), lower respiratory tract infections including pneumonia (41 [17·0%]), sepsis (33 [13·7%]), undernutrition (17 [7·1%]), and diarrhoea (nine [3·7%]; table 3). In the 425 deaths that occurred in the community with a verbal autopsy performed, the most common causes of death were malaria (103 [24·2%]); lower respiratory tract infections, including pneumonia (76 [17·9%]); other infectious diseases (73 [17·2%]); and diarrhoea (54 [12·7%]; table 3). A similar pattern of causes of death was observed for in-hospital deaths without medical records with a verbal autopsy performed (table 3). In a subset of in-hospital deaths, clinical records were incomplete for clinician-based ICD-10 cause of death assignment, and were therefore classified using the verbal autopsy instrument. In these cases, the available documentation did not allow us to establish consistently whether the child was alive on arrival or died shortly after admission. Being fully immunised was associated with a significantly lower risk of death compared with not being fully immunised (adjusted HR 0·65 [95% CI 0·55–0·76]; p<0·0001). The risk of death did not vary by sex (1·02 [0·90–1·17]; p=0·72) or season (0·96 [0·84–1·10]; p=0·54; table 4). The district of residence was associated with the risk of death (p<0·0001), being higher in Port Loko (1·53 [1·39–1·68]) and lower in Bombali (0·84 [0·76–0·93]) and Tonkolili (0·78 [0·69–0·88]) districts compared with the grand mean. Children who received sulfadoxine–pyrimethamine for malaria prevention through the EPI programme according to age had a significantly lower risk of death compared with children who did not (0·60 [0·51–0·71]; p<0·0001; table 4). Being fully immunised for age was associated with a substantial mortality reduction compared with not being fully immunised: 44% lower hazard of post-discharge death (adjusted HR 0·56 [95% CI 0·33–0·92]), 35% lower hazard of overall death (adjusted HR 0·65 [0·55–0·76]), and 29% lower odds of in-hospital death (adjusted OR 0·71 [0·54–0·95]; Table 4, Table 5, Table 6).

**Table 3:** Causes of in-hospital and community deaths

	In-hospital deaths using clinical records (n=241)	In-hospital deaths using verbal autopsy (n=74)	Community deaths using verbal autopsy (n=425)
Malaria	122 (50·6%)	20 (27·0%)	103 (24·2%)
Lower respiratory tract infections (including pneumonia)	41 (17·0%)	16 (21·6%)	76 (17·9%)
Diarrhoea	9 (3·7%)	6 (8·1%)	54 (12·7%)
Sepsis	33 (13·7%)	9 (12·2%)	29 (6·8%)
Measles	1 (0·4%)	0	0
Meningitis or encephalitis	2 (0·8%)	5 (6·8%)	17 (4·0%)
Tuberculosis	1 (0·4%)	0	0
HIV/AIDS	0	3 (4·1%)	7 (1·6%)
Other infectious diseases	3 (1·2%)	9 (12·2%)	73 (17·2%)
Congenital anomalies	0	1 (1·4%)	31 (7·3%)
Undernutrition	17 (7·1%)	1 (1·4%)	19 (4·5%)
Injuries	5 (2·1%)	0	2 (0·5%)
Other non-infectious diseases	3 (1·2%)	4 (5·4%)	14 (3·3%)
Unspecified	4 (1·7%)	0	0

Data are n (%).

**Table 4:** Factors associated with overall mortality

	Rate estimates				Crude models∗			Adjusted model∗		
	Participants	Deaths	Person-months	Incidence rate† (95% CI)	HR (95% CI)	p value	Overall p value	HR (95% CI)	p value	Overall p value
Sex										
Male	10 644	441	160 814·28	2·74 (2·50–3·01)	1 (ref)	··	··	1 (ref)	··	··
Female	9916	418	149 723·32	2·79 (2·54–3·07)	1·02 (0·89–1·16)	0·81‡	··	1·02 (0·90–1·17)	0·72‡	
District										
Port Loko	7433	454	110 904·54	4·09 (3·73–4·49)	1·60 (1·46–1·75)	<0·0001§	<0·0001	1·53 (1·39–1·68)	<0·0001§	<0·0001
Tonkolili	4259	128	64 129·54	2·00 (1·68–2·37)	0·78 (0·69–0·89)	0·0001§	··	0·78 (0·69–0·88)	0·0001§	
Bombali	8868	277	135 503·52	2·04 (1·82–2·30)	0·80 (0·72–0·89)	<0·0001§	··	0·84 (0·76–0·93)	0·0008§	
Season										
Dry	20 427	423	150 280·99	2·81 (2·56–3·10)	1 (ref)	··	··	1 (ref)	··	··
Rainy	20 405	436	160 256·61	2·72 (2·48–2·99)	0·97 (0·85–1·10)	0·62‡	··	0·96 (0·84–1·10)	0·54‡	··
Fully immunised¶										
No	20 414	353	81 917·80	4·31 (3·88–4·78)	1 (ref)	··	··	1 (ref)	··	··
Yes	20 048	506	228 619·80	2·21 (2·03–2·41)	0·46 (0·40–0·53)	<0·0001‡	··	0·65 (0·55–0·76)	<0·0001‡	··
Sulfadoxine–pyrimethamine complete||										
No	20 446	470	122 982·43	3·82 (3·49–4·18)	1 (ref)	··	··	1 (ref)	··	··
Yes	20 435	389	187 555·16	2·07 (1·88–2·29)	0·46 (0·40–0·53)	<0·0001‡	··	0·60 (0·51–0·71)	<0·0001‡	··
Total	20 560	859	310 537·60	2·77 (2·59–2·96)	··	··	··	··	··	··

Adjusted estimates are derived from a multivariable model including all covariates presented in the table. For Cox models, child age was used as the underlying time scale and was therefore not included as a separate covariate. Season was defined according to month of hospital admission or death (rainy season: May–October; dry season: November–April). p value of 0·05 or lower is considered statistically significant. HR=hazard ratio.

∗ Cox proportional hazards model.

† Per 1000 person-months at risk.

‡ Coefficients compared as difference from the reference (base) level.

§ Coefficients compared as difference from the grand mean.

¶ Fully immunised: all age-eligible Expanded Programme on Immunisation vaccines received.

|| Sulfadoxine–pyrimethamine complete: all age-eligible sulfadoxine–pyrimethamine doses received.

**Table 5:** Factors associated with in-hospital mortality

	Hospital death		Crude models∗			Adjusted model∗		
	No (n=2695)	Yes (n=241)	OR (95% CI)	p value	Overall p value	OR (95% CI)	p value	Overall p value
Age at admission								
<6 months	730 (90·9%)	73 (9·1%)	1 (ref)	··	0·11	1 (ref)	··	0·064
6 months to <12 months	1177 (91·2%)	113 (8·8%)	0·96 (0·71–1·29)	0·78†	··	0·96 (0·70–1·31)	0·79†	··
≥12 months	788 (93·5%)	55 (6·5%)	0·70 (0·49–1·00)	0·050†	··	0·66 (0·46–0·96)	0·032†	··
Sex								
Male	1535 (92·0%)	133 (8·0%)	1 (ref)	··	0·59	1 (ref)	··	0·36
Female	1160 (91·5%)	108 (8·5%)	1·08 (0·83–1·40)	0·59†	··	1·13 (0·87–1·48)	0·36†	··
District								
Port Loko	728 (88·7%)	93 (11·3%)	1·46 (1·22–1·76)	0·0001‡	<0·0001	1·46 (1·21–1·77)	0·0001‡	<0·0001
Tonkolili	940 (94·8%)	52 (5·2%)	0·64 (0·51–0·79)	0·0001‡	··	0·62 (0·49–0·77)	<0·0001‡	··
Bombali	1027 (91·5%)	96 (8·5%)	1·07 (0·89–1·29)	0·45‡	··	1·11 (0·92–1·33)	0·29‡	··
Season								
Dry	1387 (91·2%)	133 (8·8%)	1 (ref)	··	··	1 (ref)	··	··
Rainy	1308 (92·4%)	108 (7·6%)	0·86 (0·66–1·12)	0·27†	··	0·85 (0·65–1·12)	0·24†	··
Fully immunised§								
No	678 (89·4%)	80 (10·6%)	1 (ref)	··	··	1 (ref)	··	··
Yes	2017 (92·6%)	161 (7·4%)	0·67 (0·51–0·89)	0·0049†	··	0·71 (0·54–0·95)	0·021†	··
Admission type								
First admission	2688 (91·9%)	237 (8·1%)	1 (ref)	··	··	1 (ref)	··	··
Readmission	7 (63·6%)	4 (36·4%)	6·79 (1·85–24·91)	0·0039†	··	6·75 (2·00–22·75)	0·0021†	··
Time from symptom to admission, days								
0	242 (92·7%)	19 (7·3%)	1 (ref)	··	0·22	1 (ref)	··	0·22
1–2	1216 (92·1%)	105 (7·9%)	1·08 (0·65–1·79)	0·77†	··	1·17 (0·70–1·97)	0·54†	··
>2	1133 (91·8%)	101 (8·2%)	1·11 (0·67–1·85)	0·68†	··	1·20 (0·71–2·04)	0·49†	··
Undetermined	104 (86·7%)	16 (13·3%)	1·96 (0·96–4·00)	0·063†	··	2·11 (1·02–4·38)	0·045†	··
Axillary temperature on admission								
<37·5°C	1270 (92·6%)	102 (7·4%)	1 (ref)	··	··	1 (ref)	··	··
≥37·5°C	1305 (92·0%)	114 (8·0%)	1·09 (0·83–1·43)	0·55†	··	1·07 (0·81–1·42)	0·64†	··
Unknown	120 (82·8%)	25 (17·2%)	2·62 (1·61–4·29)	0·0001†	··	2·47 (1·51–4·06)	0·0004†	··
Underweight (weight-for-age Z score <–2)								
No	1884 (92·8%)	147 (7·2%)	1 (ref)	··	··	1 (ref)	··	··
Yes	811 (89·6%)	94 (10·4%)	1·49 (1·14–1·95)	0·0037†	··	1·60 (1·21–2·12)	0·0010†	··

Adjusted estimates are derived from a multivariable model including all covariates presented in the table. Season was defined according to month of hospital admission or death (rainy season: May–October; dry season: November–April). p value of 0·05 or lower is considered statistically significant. OR=odds ratio.

∗ Penalised maximum likelihood logistic regression; variance estimated by subject-cluster bootstrap (1000 replications).

† Coefficients compared as difference from the reference (base) level.

‡ Coefficients compared as difference from the grand mean.

§ Fully immunised: all age-eligible Expanded Programme on Immunization vaccines received.

**Table 6:** Factors associated with post-discharge mortality

	Rate estimates				Crude models∗			Adjusted model∗		
	Admissions	Deaths	Person-months	Incidence Rate† (95%CI)	HR (95% CI)	p value	Overall p value	HR (95% CI)	p value	Overall p value
Age at admission										
<6 months	726	28	641·78	43·63 (30·12–63·19)	1 (ref)	··	0·22	1 (ref)	··	0·45
6 months to <12 months	1164	34	1039·01	32·72 (23·38–45·80)	0·86 (0·19–3·97)	0·85‡	··	1·09 (0·24–4·99)	0·91‡	··
≥12 months	756	16	626·61	25·53 (15·64–41·68)	0·04 (0·00–1·79)	0·096‡	··	0·04 (0·00–8·41)	0·24‡	··
Sex										
Male	1501	52	1302·17	39·93 (30·43–52·41)	1 (ref)	··	··	1 (ref)	··	··
Female	1145	26	1005·23	25·86 (17·61–37·99)	0·65 (0·41–1·04)	0·071‡	··	0·64 (0·40–1·02)	0·061‡	··
District										
Port Loko	713	35	617·24	56·70 (40·71–78·98)	1·75 (1·29–2·38)	0·0003§	0·0008	1·75 (1·26–2·42)	0·0008§	0·0029
Tonkolili	918	15	804·51	18·64 (11·24–30·93)	0·57 (0·39–0·83)	0·0031§	··	0·60 (0·39–0·90)	0·015§	··
Bombali	1015	28	885·66	31·61 (21·83–45·79)	1·00 (0·73–1·37)	0·99§	··	0·96 (0·67–1·36)	0·81§	··
Season										
Dry	1545	42	1173·06	35·80 (26·46–48·45)	1 (ref)	··	··	1 (ref)	··	··
Rainy	1507	36	1134·34	31·74 (22·89–44·00)	0·91 (0·58–1·42)	0·67‡	··	0·89 (0·56–1·41)	0·61‡	··
Fully immunised¶										
No	1057	28	568·29	49·27 (34·02–71·36)	1 (ref)	··	··	1 (ref)	··	··
Yes	2205	50	1739·11	28·75 (21·79–37·93)	0·44 (0·26–0·73)	0·0016‡	··	0·56 (0·33–0·92)	0·024‡	··
Time from symptom to admission, days										
0	236	4	208·75	19·16 (7·19–51·05)	1 (ref)	··	0·22	1 (ref)	··	0·26
1–2	1196	34	1047·80	32·45 (23·19–45·41)	1·77 (0·63–4·99)	0·28‡	··	1·52 (0·52–4·49)	0·45‡	··
>2	1110	34	962·89	35·31 (25·23–49·42)	1·95 (0·69–5·49)	0·20‡	··	1·66 (0·57–4·80)	0·35‡	··
Undetermined	104	6	87·96	68·21 (30·65–151·83)	3·73 (1·04–13·40)	0·044‡	··	3·33 (0·90–12·31)	0·071‡	··
Hospital duration, days										
0–1	159	5	137·89	36·26 (15·09–87·11)	1 (ref)	··	0·0002	1 (ref)	··	0·0006
2–3	1285	20	1127·53	17·74 (11·44–27·49)	0·47 (0·18–1·27)	0·14‡	··	0·51 (0·18–1·41)	0·19‡	··
>3	1202	53	1041·97	50·87 (38·86–66·58)	1·38 (0·55–3·46)	0·49‡	··	1·44 (0·55–3·72)	0·46‡	··
Diagnosis at discharge										
Malaria	1433	42	1246·81	33·69 (24·89–45·58)	1 (ref)	··	0·30	1 (ref)	··	0·80
Lower respiratory tract infections	444	15	392·53	38·21 (23·04–63·39)	0·94 (0·53–1·66)	0·82‡	··	1·13 (0·62–2·06)	0·68‡	··
Diarrhoea	259	6	229·47	26·15 (11·75–58·20)	0·67 (0·28–1·60)	0·37‡	··	0·76 (0·31–1·89)	0·56‡	··
Undernutrition	194	9	163·65	54·99 (28·61–105·70)	1·66 (0·82–3·37)	0·16‡	··	1·16 (0·56–2·43)	0·68‡	··
Others	316	6	274·93	21·82 (9·80–48·58)	0·58 (0·24–1·39)	0·22‡	··	0·69 (0·27–1·75)	0·44‡	··
Total	2646	78	2307·40	33·80 (27·08–42·20)	··	··	··	··	··	··

Adjusted estimates are derived from a multivariable model including all covariates presented in the table. For Cox models, child age was used as the underlying time scale and was therefore not included as a separate covariate. Season was defined according to month of hospital admission or death (rainy season: May–October; dry season: November–April). p value of 0·05 or lower is considered statistically significant. HR=hazard ratio.

∗ Cox proportional hazards model.

† Per 1000 person-months at risk.

‡ Coefficients compared as difference from the reference (base) level.

§ Coefficients compared as difference from the grand mean.

¶ Fully immunised: all age-eligible Expanded Programme on Immunization vaccines received.

In competing-risks models for malaria-specific mortality, district of residence, season, and receipt of all age-eligible sulfadoxine–pyrimethamine doses were independently associated with malaria mortality. The risk was highest in Port Loko and lowest in Tonkolili, was higher during the rainy season than the dry season, and was lower in children who had received all age-eligible sulfadoxine–pyrimethamine doses than in those who had not (appendix 1 p 5). Full immunisation was not associated with malaria-specific mortality after adjustment (appendix 1 p 5).

In sensitivity analyses for malaria-specific mortality, the associations with district of residence and receipt of all age-eligible sulfadoxine–pyrimethamine doses remained consistent across alternative assumptions regarding deaths with unknown cause, and sex was not associated with malaria-specific mortality in any scenario (appendix 1 p 6). The associations with full immunisation and season varied in magnitude across scenarios, but the overall pattern of results was similar (appendix 1 p 6).

Among 2936 hospital admissions in 2517 children, 241 (8·2%) in-hospital deaths were recorded (figure 1). In adjusted logistic regression with Firth’s correction, older age at admission (≥12 months) was associated with lower risk of in-hospital death compared with children younger than 6 months (adjusted OR 0·66 [95% CI 0·46–0·96]; p=0·032). Children who were underweight at admission (weight-for-age Z score <–2) had a significantly higher risk of in-hospital death than children who were not underweight (1·60 [1·21–2·12]; p=0·0010). Median weight-for-age Z score was –1·34 (IQR –2·49 to –0·25) in hospital admissions with anthropometric data (2582 [88%] of 2936).

Being fully immunised was associated with a significantly lower risk of in-hospital mortality than not being fully immunised (adjusted OR 0·71 [95% CI 0·54–0·95]; p=0·021). Readmissions carried a substantially higher risk of in-hospital mortality compared with first admissions (6·75 [2·00–22·75]; p=0·0021; table 5). The risk of in-hospital death was highest in Port Loko district (1·46 [1·21–1·77]) and lowest in Tonkolili district (0·62 [0·49–0·77]) compared with the grand mean.

2286 children had at least one admission and were discharged alive, accounting for 2646 admissions with a post-discharge period; 78 deaths occurred during post-discharge follow-up, corresponding to a post-discharge mortality of 33·8 per 1000 child-months at risk (95% CI 27·08–42·20). Of these 78 deaths, 34 (43·6%) occurred in hospital, although ICD-10 codes were available for only 21 of them for comparison with the discharge diagnosis of the immediately preceding admission. Of these 21 deaths, the ICD-10 code of the death matched with the previous hospital discharge diagnosis in nine children (five malaria, three lower respiratory infections, and one undernutrition). In the adjusted Cox regression, district of residence was significantly associated with the risk of post-discharge death (overall p=0·0029), with a higher risk of death in Port Loko compared with the grand mean (adjusted HR 1·75 [95% CI 1·26–2·42]; table 6). Being fully immunised was associated with a reduced risk of post-discharge death (adjusted HR 0·56 [0·33–0·92]; p=0·024). No significant associations were observed for sex, season, clinical presentation at admission, or length of admission (table 6). Diagnosis at discharge was not associated with post-discharge mortality (overall p=0·80; table 6). When the post-discharge period was restricted to 14 days, 59 deaths were identified. In this analysis, discharge diagnosis remained not associated with post-discharge mortality (appendix 1 p 2).

Extending the lag period to 14 days and 21 days resulted in small increases in the number of non-incident hospitalisations (21 [0·8%] of 2695 and 30 [1·1%] of 2695, respectively, compared with seven [0·3%] of 2695 using the 7-day lag), indicating minimal effect on the overall morbidity estimates.

The geographical distribution of aggregated child mortality within the study catchment areas visualised on a 2 km hexagonal grid is shown in figure 2. Half of the participants lived farther than 1·5 km from the nearest health facility (median 1·46 km [IQR 0·84–2·53]); the distance participants lived from the nearest health facility was significantly higher in Port Loko (2·13 km [IQR 0·84–15·71]) than in Tonkolili (1·39 km [0·74–2·41]) and Bombali (1·34 km [0·89–1·86]; p<0·0001).

**Figure 2:**
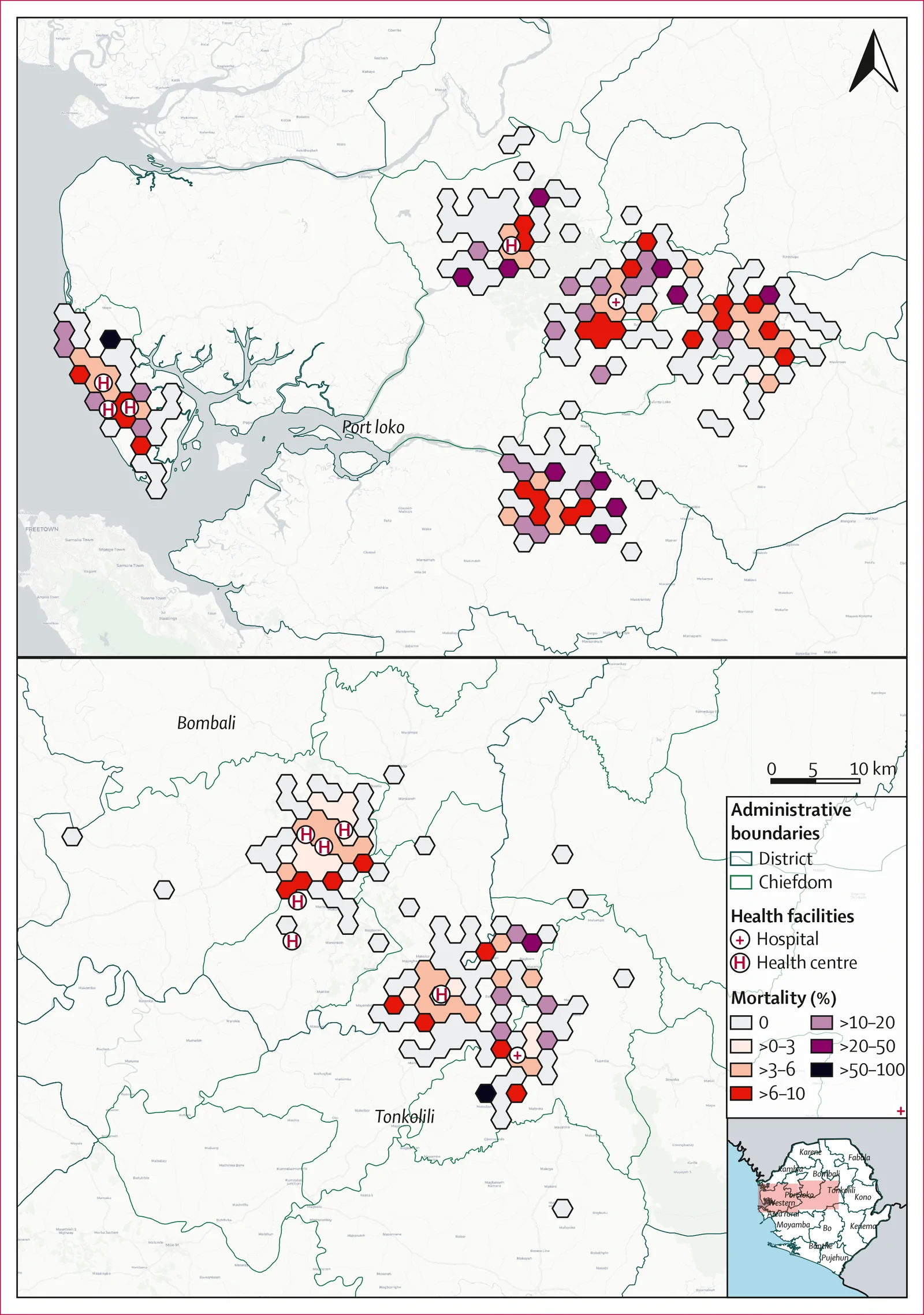
Geographical distribution of child mortality in study participants across the three study districts in Sierra Leone Mortality was mapped by 2 km grid cells based on the proportion of deaths in study participants living in each cell, calculated as the number of deaths divided by the total number of enrolled participants living in the same cell. Cells in the highest mortality category (darker shades) might reflect very small denominators rather than a high absolute number of deaths. Locations of government health facilities (hospitals and health centres) are overlaid to enable visual comparison between mortality patterns and health facility distribution.

## Discussion

This study provides crucial insights into severe morbidity and mortality in children younger than 2 years in Sierra Leone, addressing a major gap in national datasets. Infectious diseases—particularly malaria, respiratory infections, diarrhoeal illnesses, and sepsis—remain the leading causes of hospital admissions and deaths, consistent with previous data from Sierra Leone and other countries in the region.^16^^,^^32^^,^^33^ Most deaths were potentially preventable, highlighting the urgent need for implementing targeted interventions.

514 (60%) of 859 of child deaths occurred at home, aligning with findings from other rural sub-Saharan Africa settings where approximately half of deaths in children younger than 5 years take place outside health facilities.^34^ The InSilicoVA tool was used to assign the cause of death for both community deaths and in-hospital deaths without clinical records—a third of hospital deaths—often reflecting deaths occurring shortly after admission. This underscores the importance of high-quality care immediately on hospital presentation, as early mortality has been reported elsewhere; for instance, 76% of hospital deaths in Angola occurred within 24 h of admission.^35^

As with other verbal autopsy tools, the performance of InSilicoVA is limited for causes with overlapping symptom profiles, such as malaria, pneumonia, or sepsis, and thus, the distribution of cause-specific mortality from the verbal autopsy tool should be interpreted with caution. In particular, the verbal autopsy has limitations in accurately distinguishing malaria from acute respiratory tract infections in young children due to overlapping clinical features, with a sensitivity of less than 50% for malaria as a cause of death in infants.^36^^,^^37^ This likely explains the low prevalence of malaria as a cause of death in community deaths (24·2%) compared with in-hospital deaths (50·6%), suggesting underestimation of malaria mortality in rural endemic sub-Saharan Africa settings. Although unlikely, preferential hospital access for children with severe malaria might contribute to this discrepancy.

In this study, malaria was responsible for more than half of in-hospital deaths with available clinical records and non-fatal hospital admissions in young children. This finding is consistent with the high malaria transmission in Sierra Leone.^13^ These data highlight the urgent need to prioritise efforts to increase the coverage of evidence-based malaria control measures,^2^^,^^38^ which is strongly reinforced by the 40% reduction in overall mortality associated with receiving all sulfadoxine–pyrimethamine doses according to age found in this study. Malaria transmission in Sierra Leone is perennial,^13^ explaining why mortality was similar across seasons. However, overall morbidity leading to hospital admissions was significantly reduced during the rainy season (p=0·0037), probably due to difficulties in accessing health care during this period.

Lower respiratory tract infections accounted for nearly one-fifth of hospital deaths (17·0%) and admissions (16·7%), underscoring their substantial contribution to child morbidity and mortality despite high vaccine coverage against *Streptococcus pneumoniae* and *Haemophilus influenzae* type B.^39^ This burden likely reflects other aetiologies, particularly viral pathogens such as respiratory syncytial virus (RSV), which are more prevalent in young infants and highlight the need to introduce maternal RSV immunisation.^40^

The proportion of community deaths attributed to diarrhoeal diseases was three times higher than that observed in hospital, probably reflecting the limited specificity of the verbal autopsy in identifying diarrhoeal disease as the cause of death in children.^41^

HIV/AIDS accounted for 1·6% of community deaths, although none of the in-hospital deaths were attributed to the disease, reflecting the low national HIV prevalence (1·4%).^14^ Despite high awareness of HIV/AIDS, uptake of voluntary testing remains low and substantial gaps in treatment coverage persist, potentially obscuring the true burden of the epidemic.^42^

The association between full immunisation for age and lower mortality highlights the importance of strengthening immunisation programmes.^28^ Children fully immunised for their age had a 35% lower overall mortality risk, 29% reduction in in-hospital mortality, and a 44% reduction in post-discharge mortality compared with those not fully immunised. The protective effect extends beyond the diseases targeted by vaccines, reflecting recognised non-specific benefits.^43^ It has been shown that BCG, diphtheria, tetanus, and polio, and measles-containing vaccines reduce all-cause mortality beyond their target diseases.^44^

The post-discharge period is recognised as one of high vulnerability.^45^ In this study, 78 post-discharge deaths were identified, corresponding to a prevalence of 3%, which is within the 1–10% range reported from sub-Saharan Africa.^46^ In five (24%) of the 21 post-discharge deaths with clinical diagnosis, malaria was the cause of death, coinciding with the discharge diagnosis, underscoring the need to implement post-discharge malaria chemoprevention in this setting to prevent malaria-related deaths and readmissions.^47^ Consistent with previous reports from Sierra Leone and similar countries, children older than 12 months had a reduced risk of in-hospital mortality compared with infants younger than 6 months.^48^, ^49^, ^50^

Readmission after a previous hospitalisation was the strongest predictor of in-hospital death, suggesting that these children require closer post-discharge monitoring and follow-up. This finding aligns with previous evidence showing increased mortality risk in children who are readmitted to hospital.^51^ Enhanced follow-up and nutritional support are crucial for underweight children and those with recurrent hospital attendances. Undernutrition also emerged as a key risk factor for in-hospital mortality.^52^ Moreover, the small proportion of admissions attributed to undernutrition likely underestimates its true burden, since malnutrition often contributes to other conditions without being recorded as the primary diagnosis.^53^

In this study, the median distance to a health facility was 1·46 km, with significantly greater distances observed in Port Loko district. This might explain the higher mortality rates and the lower incidence of hospital admissions in Port Loko district compared with the other districts, likely due to reduced accessibility, which might warrant further investigation to inform targeted interventions in this district. This finding is aligned with evidence suggesting that greater travel distance to health facilities was associated with delayed care-seeking and increased child mortality.^54^

This study might be limited by the fact that all children included in the analysis were enrolled in a clinical trial. Although the trial remained blinded at the time of this analysis, participation in a trial might have influenced follow-up intensity, care-seeking behaviour, and outcome ascertainment compared with routine practice. Consequently, the standard of care for our participants might not fully represent that of children outside a trial setting. Nonetheless, this could suggest that the outcomes reported likely reflect better health conditions than those observed in routine care.

Because we did not collect data on caregiver behaviour, care-seeking pathways, or health-care barriers, our ability to explain why most deaths occurred in the community is limited.

Another limitation of the study is that it was conducted in three districts in northern Sierra Leone. Although the cohort was large and included both hospital and community outcomes, caution is needed when extrapolating findings to other regions of Sierra Leone. Additionally, the cohort might not fully represent all infants living in the catchment area of the health facilities, and the absence of household enumeration precluded estimation of the full source birth-cohort in each community.

This study was done in a country that had experienced a devastating civil war that destroyed health infrastructure, displaced populations, and decimated the health workforce. The 2014–16 Ebola virus disease epidemic further strained the health system.^55^ This study, conducted 5–8 years after the Ebola virus disease epidemic, reflects a health system still recovering from these crises. The challenges we observed must be understood in this context. The resilience of the health system and the progress made, including the nation-wide perennial malaria chemoprevention and malaria vaccine implementations are notable achievements. However, substantial gaps remain, particularly regarding data system strengthening and surveillance and management of child mortality and morbidity. Sustained international support for health system strengthening in post-conflict and post-epidemic settings remains essential for achieving child survival goals.

Few studies comprehensively document disease and mortality in young children in rural Africa, even in settings that apply evidence-based tools, such as Sierra Leone. Despite a time when global health received exceptional attention and investment—with substantial international funding and a capable Ministry of Health implementing proven interventions in Sierra Leone—child mortality remains high. Infectious diseases continue to be the leading causes of death. This stark reality underscores the unfinished global health agenda and the urgent need for implementing more effective tools and strategies.

## Data sharing

All data used in this Article will be made available on request in compliance with the institutional policies. As the dataset contains potentially identifying information on participants, it is stored under restricted access. For more detailed information beyond the information provided, there is a process of managed access requiring the submission of a request for consideration. Please contact this email for data access: andreu.bofill@isglobal.org.

## Declaration of interests

We declare no competing interests.
