# Supplementary material for: Causes of mortality and severe morbidity in young children in Sierra Leone: a prospective cohort study
Source: Lancet Glob Health. 2026 Aug 10;14(10):None. doi: 10.1016/j.langlo.2026.104025 (PMC13652798; doi:10.1016/j.langlo.2026.104025)
Supplement: Supplementary file 1 [file mmc1.pdf]

THE LANCET
Global Health

## Supplementary appendix 1

This appendix formed part of the original submission and has been peer reviewed.
We post it as supplied by the authors.

Supplement to: Owusu-Kyei K, Cussó M, Fombah AE, et al. Causes of mortality and severe morbidity in young children in Sierra Leone: a prospective cohort study. *Lancet Glob Health* 2026. https://doi.org/10.1016/j.langlo.2026.104025

**Supplementary Table S1.** Age-disaggregated cause patterns.

| Diagnosis | | Age | | | Total |
|---|---|---|---|---|---|
| | | < 6m | 6-<12m | >= 12m | |
| Hospital admissions (non-fatal) [1] | Malaria | 268 (37%) | 678 (58%) | 513 (66%) | 1459 (54%) |
| | Lower respiratory infections | 249 (34%) | 140 (12%) | 60 (8%) | 449 (17%) |
| | Diarrhoea | 76 (10%) | 128 (11%) | 55 (7%) | 259 (10%) |
| | Undernutrition | 26 (4%) | 85 (7%) | 87 (11%) | 198 (7%) |
| | Sepsis | 47 (6%) | 66 (6%) | 15 (2%) | 128 (5%) |
| | Injuries | 26 (4%) | 24 (2%) | 23 (3%) | 73 (3%) |
| | Other infectious disease | 23 (3%) | 23 (2%) | 11 (1%) | 57 (2%) |
| | Other non-infectious disease | 8 (1%) | 6 (1%) | 14 (2%) | 28 (1%) |
| | Meningitis | 2 (0%) | 8 (1%) | 1 (0%) | 11 (0%) |
| | Unspecified | 3 (0%) | 6 (1%) | 2 (0%) | 11 (0%) |
| | Measles | 2 (0%) | 6 (1%) | 2 (0%) | 10 (0%) |
| | Tuberculosis | 0 (0%) | 3 (0%) | 0 (0%) | 3 (0%) |
| | HIV | 0 (0%) | 2 (0%) | 0 (0%) | 2 (0%) |
| | *Total* | *730 (100%)* | *1175 (100%)* | *783 (100%)* | *2688 (100%)* |
| Cause of death [1] | Malaria | 49 (22%) | 114 (35%) | 82 (42%) | 245 (33%) |
| | Lower respiratory infections | 58 (26%) | 45 (14%) | 30 (16%) | 133 (18%) |
| | Other infectious disease | 23 (10%) | 35 (11%) | 27 (14%) | 85 (11%) |
| | Sepsis | 34 (15%) | 28 (9%) | 9 (5%) | 71 (10%) |
| | Diarrhoea | 19 (8%) | 36 (11%) | 14 (7%) | 69 (9%) |
| | Undernutrition | 4 (2%) | 22 (7%) | 11 (6%) | 37 (5%) |
| | Congenital anomalies | 18 (8%) | 11 (3%) | 3 (2%) | 32 (4%) |
| | Meningitis/encephalitis | 5 (2%) | 13 (4%) | 6 (3%) | 24(3%) |
| | Other non-infectious disease | 5 (2%) | 10 (3%) | 3 (2%) | 18 (2%) |
| | HIV/AIDS | 1 (0%) | 5 (2%) | 4 (2%) | 10 (1%) |
| | Injuries | 4 (2%) | 1 (0%) | 2 (1%) | 7 (1%) |
| | Unspecified | 0 (0%) | 3 (1%) | 1 (1%) | 4 (1%) |
| | Other non-infectious disease | 3 (1%) | 0 (0%) | 0 (0%) | 3 (0%) |
| | | | | | |
| | Tuberculosis | 1 (0%) | 0 (0%) | 0 (0%) | 1 (0%) |
| | Measles | 0 (0%) | 0 (0%) | 1 (1%) | 1 (0%) |
| | *Total* | *224 (100%)* | *323 (100%)* | *193 (100%)* | *740 (100%)* |

1: Frequency (Column percentage)

**Supplementary Table S2.** Factors associated with post-discharge mortality when the post-discharge period was defined as 14 days after hospital discharge.

| | | Rate estimates | | | | Crude Models [1] | | | Adjusted Model [1] | | |
|---|---|---|---|---|---|---|---|---|---|---|---|
| | | Admissions | Deaths | Person-months | Incidence Rate [2] (95%CI) | Hazard Ratio (95%CI) | p-value | Overall p-value | Hazard Ratio (95%CI) | p-value | Overall p-value |
| Age at admission | < 6m | 726 | 23 | 325.95 | 70.56 (46.89-106.18) | 1.00 | - | 0.0003 | 1.00 | - | < 0.0001 |
| | 6-<12m | 1164 | 25 | 526.38 | 47.49 (32.09-70.29) | 1.04 (0.20-5.48) | 0.9637 [a] | | 1.62 (0.26-10.00) | 0.6015 [a] | |
| | >= 12m | 756 | 11 | 330.99 | 33.23 (18.40-60.01) | 0.02 (0.00-0.25) | 0.0024 [a] | | 0.01 (0.00-0.17) | 0.0012 [a] | |
| Sex | Male | 1501 | 41 | 670.07 | 61.19 (45.05-83.10) | 1.00 | - | - | 1.00 | - | - |
| | Female | 1145 | 18 | 513.26 | 35.07 (22.10-55.66) | 0.58 (0.33-1.00) | 0.0512 [a] | | 0.53 (0.30-0.93) | 0.0271 [a] | |
| District | Port Loko | 713 | 27 | 316.41 | 85.33 (58.52-124.43) | 1.84 (1.29-2.63) | 0.0008 [b] | 0.0014 | 1.98 (1.33-2.95) | 0.0007 [b] | 0.0016 |
| | Tonkolili | 918 | 9 | 411.45 | 21.87 (11.38-42.04) | 0.47 (0.29-0.75) | 0.0014 [b] | | 0.42 (0.25-0.72) | 0.0018 [b] | |
| | Bombali | 1015 | 23 | 455.46 | 50.50 (33.56-75.99) | 1.16 (0.80-1.67) | 0.4296 [b] | | 1.20 (0.79-1.82) | 0.3943 [b] | |
| Season | Dry | 1471 | 35 | 605.86 | 57.77 (41.48-80.46) | 1.00 | - | - | 1.00 | - | - |
| | Rainy | 1413 | 24 | 577.47 | 41.56 (27.86-62.01) | 0.72 (0.43-1.22) | 0.2243 [a] | | 0.70 (0.41-1.22) | 0.2118 [a] | |
| Fully immunised | No | 888 | 20 | 291.84 | 68.53 (44.21-106.22) | 1.00 | - | - | 1.00 | - | - |
| | Yes | 2143 | 39 | 891.48 | 43.75 (31.96-59.88) | 0.48 (0.27-0.83) | 0.0094 [a] | | 0.65 (0.36-1.16) | 0.1404 [a] | |
| Symptom–admission delay (days) | 0 | 236 | 3 | 106.15 | 28.26 (9.11-87.63) | 1.00 | - | 0.3773 | 1.00 | - | 0.3917 |
| | 1-2 | 1196 | 25 | 536.81 | 46.57 (31.47-68.92) | 1.71 (0.51-5.78) | 0.3849 [a] | | 1.41 (0.39-5.08) | 0.5953 [a] | |
| | >2 | 1110 | 27 | 494.93 | 54.55 (37.41-79.55) | 2.00 (0.59-6.74) | 0.2657 [a] | | 1.66 (0.47-5.88) | 0.4302 [a] | |
| | Undetermined | 104 | 4 | 45.43 | 88.05 (33.05-234.61) | 3.61 (0.79-16.57) | 0.0985 [a] | | 3.41 (0.69-16.83) | 0.1329 [a] | |
| Hospital duration (days) | 0-1 | 159 | 4 | 70.53 | 56.72 (21.29-151.12) | 1.00 | - | 0.0023 | 1.00 | - | 0.0044 |
| | 2-3 | 1285 | 15 | 577.53 | 25.97 (15.66-43.08) | 0.43 (0.14-1.29) | 0.1323 [a] | | 0.50 (0.16-1.56) | 0.2361 [a] | |
| | >3 | 1202 | 40 | 535.26 | 74.73 (54.82-101.88) | 1.23 (0.44-3.41) | 0.6896 [a] | | 1.42 (0.48-4.16) | 0.5277 [a] | |
| Diagnose at discharge | Malaria | 1433 | 31 | 640.76 | 48.38 (34.02-68.79) | 1.00 | - | 0.7195 | 1.00 | - | 0.7713 |
| | Lower respiratory infections | 444 | 12 | 199.34 | 60.20 (34.19-106.00) | 1.04 (0.55-1.98) | 0.8992 [a] | | 1.42 (0.74-2.73) | 0.2891 [a] | |
| | Diarrhoea | 259 | 5 | 116.94 | 42.76 (17.80-102.72) | 0.75 (0.28-2.00) | 0.5603 [a] | | 0.93 (0.33-2.64) | 0.8957 [a] | |
| | Undernutrition | 194 | 6 | 85.56 | 70.13 (31.51-156.09) | 1.46 (0.62-3.44) | 0.3813 [a] | | 1.10 (0.44-2.71) | 0.8433 [a] | |
| | Others | 316 | 5 | 140.72 | 35.53 (14.79-85.36) | 0.66 (0.26-1.71) | 0.3981 [a] | | 0.79 (0.26-2.43) | 0.6852 [a] | |
| *Total* | | 2646 | 59 | 1183.32 | 49.86 (38.63-64.35) | - | | | | | |

1: Cox proportional hazards model with coefficients compared as follows:

a: Differences from the reference (base) level

b: Differences from the grand mean
2: Per 1000 person-months at risk
CI: confidence interval; Fully immunised: All age-eligible EPI vaccines received

**Supplementary Table S3.** Factors associated with malaria-specific hospital admissions.

| | | Rate estimates | | | | Crude Models [1] | | | Adjusted Model [1] | | |
|---|---|---|---|---|---|---|---|---|---|---|---|
| | | Subjects | Malaria episodes | Person-months | Incidence Rate [2] (95%CI) | IRR (95%CI) | p-value | Overall p-value | IRR (95%CI) | p-value | Overall p-value |
| Age | < 6m | 20559 | 268 | 90920.87 | 2.95 (2.62-3.32) | 1.00 | - | < 0.0001 | 1.00 | - | < 0.0001 |
| | 6-<12m | 20129 | 678 | 118086.05 | 5.74 (5.33-6.19) | 1.95 (1.69-2.24) | < 0.0001 [a] | | 1.96 (1.70-2.26) | < 0.0001 [a] | |
| | >= 12m | 18481 | 513 | 101143.52 | 5.07 (4.65-5.53) | 1.72 (1.48-2.00) | < 0.0001 [a] | | 1.75 (1.49-2.05) | < 0.0001 [a] | |
| Sex | Male | 10644 | 837 | 160589.62 | 5.21 (4.87-5.58) | 1.00 | - | - | 1.00 | - | - |
| | Female | 9916 | 622 | 149560.82 | 4.16 (3.84-4.50) | 0.80 (0.71-0.89) | 0.0001 [a] | | 0.80 (0.71-0.89) | 0.0001 [a] | |
| District | Port Loko | 7433 | 505 | 110808.80 | 4.56 (4.18-4.97) | 0.97 (0.89-1.04) | 0.3849 [b] | 0.6826 | 0.97 (0.89-1.05) | 0.4064 [b] | 0.7007 |
| | Tonkolili | 4259 | 310 | 63999.29 | 4.84 (4.33-5.41) | 1.03 (0.94-1.12) | 0.5513 [b] | | 1.03 (0.94-1.12) | 0.5383 [b] | |
| | Bombali | 8868 | 644 | 135342.35 | 4.76 (4.40-5.14) | 1.01 (0.94-1.09) | 0.8230 [b] | | 1.01 (0.93-1.08) | 0.8720 [b] | |
| Season | Dry | 20426 | 761 | 150087.55 | 5.07 (4.72-5.44) | 1.00 | - | - | 1.00 | - | - |
| | Rainy | 20404 | 698 | 160062.89 | 4.36 (4.05-4.70) | 0.86 (0.78-0.95) | 0.0041 [a] | | 0.85 (0.77-0.95) | 0.0027 [a] | |
| Fully immunised | No | 20414 | 371 | 81807.29 | 4.54 (4.10-5.02) | 1.00 | - | - | 1.00 | - | - |
| | Yes | 20048 | 1088 | 228343.16 | 4.76 (4.49-5.06) | 1.05 (0.93-1.18) | 0.4198 [a] | | 0.98 (0.85-1.13) | 0.7592 [a] | |
| SP complete | No | 20446 | 592 | 122812.86 | 4.82 (4.45-5.22) | 1.00 | - | - | 1.00 | - | - |
| | Yes | 20435 | 867 | 187337.58 | 4.63 (4.33-4.95) | 0.96 (0.86-1.07) | 0.4560 [a] | | 1.03 (0.91-1.18) | 0.6292 [a] | |
| *Total* | | 20560 | 1459 | 310150.44 | 4.70 (4.47-4.95) | - | | | | | |

1: Negative Binomial Regression with coefficients compared as follows:

a: Differences from the reference (base) level

b: Differences from the grand mean

2: Per 1000 person-months at risk

CI: confidence interval; IRR: Incidence Rate Ratio; Fully immunised: All age-eligible EPI vaccines received; SP complete: All age-eligible SP doses received

**Supplementary Table S4.** Factors associated with malaria-specific mortality in competing-risks models.

| | | Rate estimates | | | | Crude Models [1] | | | Adjusted Model [1] | | |
|---|---|---|---|---|---|---|---|---|---|---|---|
| | | Subjects | Deaths | Person-months | Incidence Rate [2] (95%CI) | sHR (95%CI) | p-value | Overall p-value | sHR (95%CI) | p-value | Overall p-value |
| Sex | Male | 10644 | 129 | 160814.28 | 0.80 (0.68-0.95) | 1.00 | - | - | 1.00 | - | - |
| | Female | 9916 | 116 | 149723.32 | 0.77 (0.65-0.93) | 0.96 (0.75-1.24) | 0.7718 [a] | | 0.97 (0.75-1.25) | 0.8126 [a] | |
| District | Port Loko | 7433 | 143 | 110904.54 | 1.29 (1.09-1.52) | 1.89 (1.58-2.27) | < 0.0001 [b] | < 0.0001 | 1.83 (1.53-2.20) | < 0.0001 [b] | < 0.0001 |
| | Tonkolili | 4259 | 28 | 64129.54 | 0.44 (0.30-0.63) | 0.65 (0.50-0.85) | 0.0013 [b] | | 0.64 (0.49-0.84) | 0.0010 [b] | |
| | Bombali | 8868 | 74 | 135503.52 | 0.55 (0.43-0.69) | 0.81 (0.66-1.00) | 0.0470 [b] | | 0.85 (0.69-1.04) | 0.1202 [b] | |
| Season | Dry | 20427 | 100 | 150280.99 | 0.67 (0.55-0.81) | 1.00 | - | - | 1.00 | - | - |
| | Rainy | 20405 | 145 | 160256.61 | 0.90 (0.77-1.06) | 1.34 (1.04-1.74) | 0.0229 [a] | | 1.34 (1.04-1.73) | 0.0244 [a] | |
| Fully immunised | No | 20414 | 89 | 81917.80 | 1.09 (0.88-1.34) | 1.00 | - | - | 1.00 | - | - |
| | Yes | 20048 | 156 | 228619.80 | 0.68 (0.58-0.80) | 0.56 (0.43-0.73) | < 0.0001 [a] | | 0.88 (0.66-1.18) | 0.3951 [a] | |
| SP complete | No | 20446 | 142 | 122982.43 | 1.15 (0.98-1.36) | 1.00 | - | - | 1.00 | - | - |
| | Yes | 20435 | 103 | 187555.16 | 0.55 (0.45-0.67) | 0.44 (0.34-0.58) | < 0.0001 [a] | | 0.50 (0.38-0.66) | < 0.0001 [a] | |
| *Total* | | 20560 | 245 | 310537.60 | 0.79 (0.70-0.89) | - | | | | | |

1: Fine–Gray competing-risk model with coefficients compared as follows:
  a: Differences from the reference (base) level
  b: Differences from the grand mean
2: Per 1000 person-months at risk
SP: Sulfadoxine Pyrimethamine; sHR: subdistribution Hazard Ratio derived from the Fine-Gray model; CI: confidence interval;
Fully immunised: All age-eligible EPI vaccines received; SP complete: All age-eligible SP doses received

**Supplementary Table S5**. Sensitivity analyses for malaria-specific mortality under alternative assumptions for deaths of unknown cause.

| Covariate level | **Primary analysis** 245 malaria deaths 614 competing events **Adjusted sHR (95% CI)** | **33% randomly reclassified as malaria** 272-293 malaria deaths 566-587 competing events **Mean adjusted sHR (range across 10 runs)** | **Worst-case scenario** 364 malaria deaths 495 competing events **Adjusted sHR (95% CI)** |
|---|---|---|---|
| Female sex | 0.97 (0.75-1.25) | 0.99 (0.93-1.07) | 0.99 (0.81-1.22) |
| District: Port Loko | 1.83 (1.53-2.20) | 1.72 (1.61-1.81) | 1.55 (1.34-1.79) |
| District: Tonkolili | 0.64 (0.49-0.84) | 0.71 (0.67-0.77) | 0.79 (0.65-0.96) |
| District: Bombali | 0.85 (0.69-1.04) | 0.83 (0.79-0.87) | 0.82 (0.69-0.96) |
| Rainy season | 1.34 (1.04-1.73) | 1.25 (1.18-1.31) | 1.14 (0.93-1.40) |
| Fully immunised | 0.88 (0.66-1.18) | 0.80 (0.78-0.86) | 0.68 (0.54-0.85) |
| SP complete | 0.50 (0.38-0.66) | 0.49 (0.47-0.50) | 0.46 (0.37-0.59) |

Fully immunised: All age-eligible EPI vaccines received; SP complete: All age-eligible SP doses received

Estimates are adjusted subdistribution hazard ratios (sHRs) from Fine and Gray competing-risks models for malaria-specific mortality. In the primary analysis, deaths from causes other than malaria, including deaths of unknown cause, were treated as competing events. In the 33% scenario, 33% of deaths with unknown cause were randomly reclassified as malaria deaths, reflecting the proportion of malaria among deaths with known cause; the procedure was repeated 10 times from a fixed random seed, and results are shown as the mean adjusted sHR and range across simulations. In the worst-case scenario, all deaths with unknown cause were reclassified as malaria deaths. District effects are shown as deviations from the grand mean.

**Supplementary Figure 1.** Kaplan–Meier cumulative probability of death by age among young children in Sierra Leone. Age is shown in months. Children were recruited at 6–10 weeks of age and followed until 18 months of age. The at-risk table shows the number of children under observation and the cumulative number of deaths at each age shown. Total deaths by 18 months were 859.

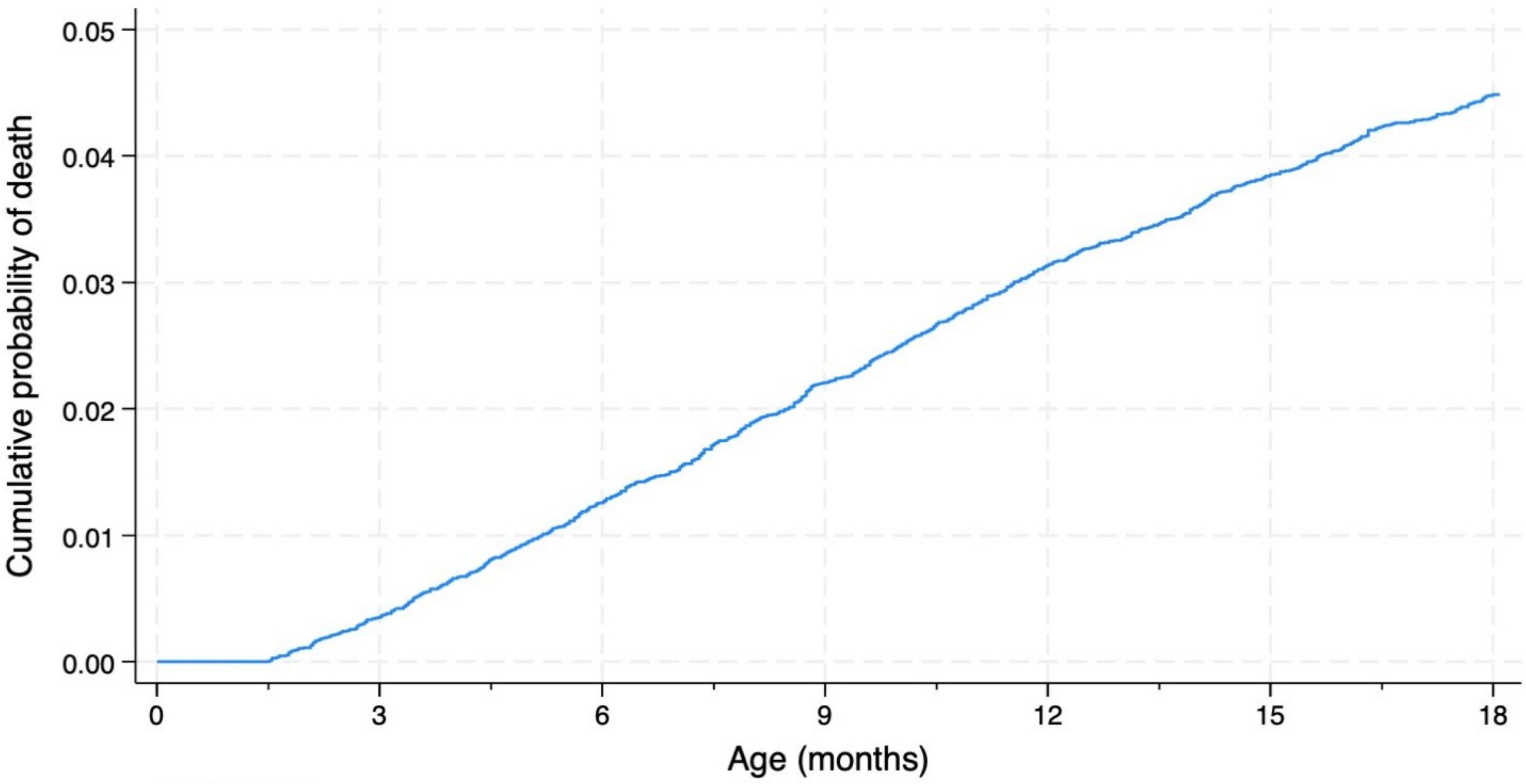


**At-risk table**

| | | | | | | |
|---|---|---|---|---|---|---|
| At risk | 20408 | 20137 | 19850 | 18566 | 16292 | 8902 |
| Cum. fail | 68 | 253 | 446 | 630 | 758 | 859 |

N = 20,560 children recruited at 6-10 weeks of age. Numbers at risk reflect children under observation at each age.

**ICARIA Safety Team**

| First name | Surname |
| --- | --- |
| Gerard | Martinez |
| Celia | Martí |
| María | Cussó |
| Emma | Wilheim |
| María | Iriondo |
| Laia | Nou |
| Noelia | Arguedas |
| María | Suay |
| Paula | Sevilla |
| Laura | Pellicer |
| Ariadna | Rubio |
| Clara | Izquierdo |
| Maria Goretti | López |
| Victoria | Fumadó |
