# Supplementary material for: Causes of mortality and severe morbidity in young children in Sierra Leone: a prospective cohort study
Source: Lancet Glob Health. 2026 Aug 10;14(10):None. doi: 10.1016/j.langlo.2026.104025 (PMC13652798; doi:10.1016/j.langlo.2026.104025)
Supplement: Supplementary file 2 [file mmc2.pdf]

THE LANCET
Global Health

Supplementary appendix 2

This appendix formed part of the original submission and has been peer reviewed. We post it as supplied by the authors.

Supplement to: Owusu-Kyei K, Cussó M, Fombah AE, et al. Causes of mortality and severe morbidity in young children in Sierra Leone: a prospective cohort study. *Lancet Glob Health* 2026. https://doi.org/10.1016/j.langlo.2026.104025

Equitable Partnership Declaration

If any questions do not apply to your study, please indicate "N/A" for "not applicable.
For more information on how to complete this form see the Information for Authors document.

Researcher considerations

1. Please detail the involvement that researchers who are based in the country or countries of study had during a) study design; b) clinical study processes, such as processing blood samples, prescribing medication, or patient recruitment; c) data interpretation; and d) manuscript preparation, commenting on all aspects. If they were not involved in any of these aspects, please explain why.

   This should include a thorough description of their leadership roles in the study. Are local researchers named in the author list or the acknowledgements, or are they not mentioned at all (and, if not, why)? Please also describe the involvement of early career researchers based in the location of the study. Some of this information might be repeated from the Contributors section in the manuscript. Note: we adhere to ICMJE authorship criteria for naming authors on a paper.

**a) Study design:**<br><br>Study design was conceived by CM, KOK and LQ. The study design was finalized through consultations with Sierra Leone Ministry of Health and College of Medicine and Allied Health Sciences (COMAHS), University of Sierra Leone project coordinators, whose input was essential for the selection of study sites and protocol development. All Sierra Leone-based senior investigators and early career researchers who fulfilled ICMJE authorship criteria are included as co-authors in the manuscript.
**b) Clinical study processes:**<br><br>All data collection processes were led by in-country research staff. The in-country research Investigators and coordinators, in particular, played a pivotal leadership role in the study participants recruitment, informed consent, follow-up visits, household surveillance, hospital surveillance, verbal autopsy interviews, clinical assessments, adverse event reporting, and and retention activities. This included liaising with the community stakeholders, local Ethics Committee, and government regulatory focal points; ensuring correct study protocol approvals and implementation; overseeing and mentoring data collection staff; supervising day to day site operations; managing procurements and supply chains; and ensuring in-country research regulations were met.<br>Field assistants and clinic health workers were recruited and trained in standardized data collection and reporting. The project also collaborated closely with district health management teams and local health facility personnel.
**c) Data interpretation:**<br><br>Researchers based in Sierra Leone reviewed study data at regular intervals throughout the study and were actively involved in interpretation of study findings. Interpretation meetings included investigators from Sierra Leone and international partner institutions to ensure that

findings were contextualised within the local epidemiological, health-system, and sociocultural setting.<br>Local researchers contributed particularly to interpretation of:<br>• malaria epidemiology,<br>• verbal autopsy findings,<br>• geographic differences in mortality,<br><br>The inclusion of Sierra Leone-based investigators was essential to ensure that the analysis conclusions and policy implications reflected the realities of the local health system and child health priorities.
**d) Manuscript preparation:**<br><br>Following agreement on the key findings, the initial manuscript was drafted by KOK, then circulated for all co-authors, including in-country authors to contribute to and provide feedback into the initial narrative draft.<br>All investigators from the Spain and Sierra Leone reviewed the manuscript and provided comments, and the revised version was reviewed and approved by all key investigators from both countries.

2. How was funding used to remunerate and enhance the skills of researchers in the countries of study? And how was funding used to improve research infrastructure at the study sites?

   Potentially effective investments into long-term skills and opportunities within local institutions could include training or mentorship in analytical techniques and manuscript writing, opportunities to lead all or specific aspects of the study, financial remuneration rather than requiring volunteers, and other professional development and educational opportunities.

   Improvements to research infrastructure could include funding extended trial designs (eg, platform trials), establishment of long-term contracts for research staff, building research facilities, and setting up local control of funding allocation.

**Skills:**<br>Study funding supported the recruitment, remuneration, and professional development of Sierra Leonean research staff, including physicians, nurses, field workers, data officers, study coordinators, and junior researchers.<br>As part of funding for this study, 120 local staff were hired and trained in the intervention strategies employed in the study as well as structured assessment skills. The study also supported 16 staffs on short term courses and 31 on long term programs (including 2 local PhD candidates).<br>Leading staff have also been mentored in manuscript writing, including assistance in first-authoring manuscripts.
**Research infrastructure:**<br>As a result of the training and skills the research staff gained through the funding of this study, many have gone on to take more senior roles on new research projects. Additionally,

due to success of the study and existing infrastructure, new research opportunities continue to come to the areas.
The study supported the local partner institution, COMAHS to acquire motorbikes and vehicles for field operations. This infrastructure will remain available for future research projects seeking to leverage the capacity built through this study.

3. How did you safeguard the researchers who implemented the study?

Please describe how you guaranteed safe working conditions for study staff, including provision of appropriate personal protective equipment, protection from violence, and prevention of overworking.

The study prioritised the safety, wellbeing, and ethical protection of all research staff. All study personnel received training in occupational health and safety procedures and were provided with personal protective equipment where required. Standard operating procedures covering all aspects of the study were developed and made readily available throughout the study period.
All staff involved in this trial were supervised throughout the trial by the lead investigators to ensure proper management of psychological and physical safety.

<u>Benefits to the communities and regions of study</u>

4. How does the study address the research and policy priorities of its location?

How were the local priorities determined and then used to inform the research question? Who decided which priorities to take forward? Which elements of the study address those priorities?

The ICARIA trial was designed to address major child survival priorities in Sierra Leone, where infectious diseases such as malaria, diarrhoea, and pneumonia, malnutrition, and limited access to healthcare remain leading contributors to under-five morbidity and mortality.
The study objectives aligned closely with Sierra Leone Ministry of Health priorities identified by:

- national malaria control program strategies;
- child survival policies; and
- Expanded Programme on Immunization (EPI) priorities.

Local health authorities, clinicians, and Sierra Leone-based investigators contributed to identifying the need for evidence regarding interventions capable of reducing childhood morbidity and mortality in high-burden settings.
The study specifically addressed:

- malaria prevention in young children;

- severe childhood morbidity surveillance;
- causes of childhood mortality;
- feasibility of integrating preventive interventions within routine EPI systems; and
- generation of locally relevant evidence to inform child health policy.

The findings are expected to contribute directly to policy discussions regarding infectious diseases prevention strategies, child survival interventions, and integrated community-based health programmes in Sierra Leone and similar high-burden settings in sub-Saharan Africa.

5. How will research products be shared in the community of study?

For instance, will you be providing written or oral layperson summaries for non-academic information sharing? Will study data be made available to institutions in the region(s) of study? The Lancet Group *encourages authors to translate the summary (abstract) into relevant languages after paper editing; do you intend to translate your summary?*

We have presented preliminary findings of the study to the national ministries of health and the three districts in the country where the study was held for initial feedback and discussion. Furthermore, once results are finalized, we do plan to include translated abstracts in the local languages designed for non-scientific audiences. Also, local community meetings whereby the local community will be invited to hear oral presentation of the results and any implications for the local communities.
The study team also intends to support access to relevant data through appropriate institutional and ethical governance mechanisms consistent with participant confidentiality and data protection requirements.

6. How were individuals, communities, and environments protected from harm?

a) *How did you ensure that sensitive patient data were handled safely and respectfully? Was there any potential for stigma or discrimination against participants arising from any of the procedures or outcomes of the study?*

Participant confidentiality and data protection were maintained throughout the study.
All patient files and data collection forms were maintained securely in a locked file throughout the study to ensure participant confidentiality
All study participants were assigned unique identification numbers, and personally identifiable information such as child's name and address were stored separately from analytical datasets.
Electronic data systems were password-protected and accessible only to authorised study personnel.
Ethical approvals and informed consent procedures included clear explanations regarding confidentiality, voluntary participation, and data use.
here was no potential for discrimination towards participants. No participant groups were

singled out on the basis of ethnicity, religion, socioeconomic status, or disease condition.

b) *Might any of the tests be experienced as invasive or culturally insensitive?*

All tests required in the study were validated and tested to be culturally appropriate prior to the study. Sample collection (nasal swabs, rectal swabs, venous blood sample, and dried blood spots) are invasive and caregivers were counselled on these procedures and only those who consented were enrolled.

c) *How did you determine that work was sensitive to traditions, restrictions, and considerations of all cultural and religious groups in the study population?*

During the study design phase and throughout the implementation of the study, community leaders, local health workers, community stakeholders, and local government officials were consulted to obtain valuable insights and guidance on cultural and religious sensitivities that needed to be considered. This collaboration allowed the researchers to gain a deeper understanding of the cultural nuances and specific considerations of the study population. This included guidance on using appropriate language, visuals, and channels of communication to effectively reach and engage different cultural and religious groups
Consent materials and participant information were explained in locally understood languages where necessary. Community engagement activities were conducted before and during implementation to strengthen trust, transparency, and acceptability.
The feedback and advice from these consultations was directly applied throughout the implementation of the study.

d) *Were biowaste and radioactive waste disposed of in accordance with local laws?*

Yes, clinical and laboratory waste generated during the study was handled and disposed of in accordance with local laws and policies related to appropriate disposal requirements for any biowaste associated with the study.
No radioactive materials were used during the study.

e) *Were any structures built that would have impacted members of the community or the environment (such as handwashing facilities in a public space)? If so, how did you ensure that you had appropriate community buy-in?*

NA

f) *How might the study have impacted existing health-care resources (such as staff workloads, use of equipment that is typically employed elsewhere, or reallocation of public funds)?*

The study was designed to minimise strain on existing healthcare services by integrating activities within routine health systems where feasible.
Additional study personnel including medical doctors, nurses, field workers, community mobilisers, and laboratory technicians were hired by the study to provide support to all participating facilities to reduce excess workload on routine healthcare staff.
The project also strengthened surveillance and data collection systems that supported local healthcare delivery and public health monitoring.

7. Confirm that local ethics review was sought, and please provide the approval number. If not sought, please explain why.

Ethical approval for the study was obtained from the Sierra Leone Ethics and Scientific Review Committee (dated August 9, 2020, no approval number) and the Hospital Clínic of Barcelona Research Ethics Committee (Registration No: HCB/2020/0173, dated August 28, 2020) prior to study implementation.<br>The study was conducted in accordance with the principles of the Declaration of Helsinki and Good Clinical Practice guidelines.

<u>Secondary analyses</u>

8. Have the data analysed in your study been extracted from another source, such as a national survey, rather than being directly collected by the authors of this paper?

No

If the authors of this paper were not involved in data collection, how were the findings interpreted with sufficient contextual knowledge?
The Lancet Group *believe contextual understanding is crucial for informed data analysis and interpretation*.

All authors based in Sierra Leone were involved in the study data collection. Authors of this paper who were not directly involved in data collection were in close contact with members of the site study teams to provide local contextual expertise to aid in data interpretation.

---

9. Please provide the title (eg, Dr/Prof, Mr/Mrs/Ms/Mx), name, and email address of an author who can be contacted about this statement.

**Name:** Mr. Kwabena Owusu-Kyei<br>**Email:** Kwabena.owusukyei@isglobal.org

10. Finally, please provide the title and name of an author from one country of study who has seen and approved this form.

**Name:** Prof. Mohamed Samai, PhD
